# Association between Image Characteristics on Chest CT and Severe Pleural Adhesion during Lung Cancer Surgery

**DOI:** 10.1371/journal.pone.0154694

**Published:** 2016-05-12

**Authors:** Kwang Nam Jin, Yong Won Sung, Se Jin Oh, Ye Ra Choi, Hyoun Cho, Jae-Sung Choi, Hyeon-Jong Moon

**Affiliations:** 1 Department of Radiology, Seoul Metropolitan Government-Seoul National University Boramae Medical Center, Seoul, Korea; 2 Department of Cardiothoracic Surgery, Seoul Metropolitan Government-Seoul National University Boramae Medical Center, Seoul, Korea; Peking University People Hospital, CHINA

## Abstract

The aim of this study was to investigate the association between image characteristics on preoperative chest CT and severe pleural adhesion during surgery in lung cancer patients. We included consecutive 124 patients who underwent lung cancer surgeries. Preoperative chest CT was retrospectively reviewed to assess pleural thickening or calcification, pulmonary calcified nodules, active pulmonary inflammation, extent of emphysema, interstitial pneumonitis, and bronchiectasis in the operated thorax. The extent of pleural thickening or calcification was visually estimated and categorized into two groups: localized and diffuse. We measured total size of pulmonary calcified nodules. The extent of emphysema, interstitial pneumonitis, and bronchiectasis was also evaluated with a visual scoring system. The occurrence of severe pleural adhesion during lung cancer surgery was retrospectively investigated from the electrical medical records. We performed logistic regression analysis to determine the association of image characteristic on chest CT with severe pleural adhesion. Localized pleural thickening was found in 8 patients (6.5%), localized pleural calcification in 8 (6.5%), pulmonary calcified nodules in 28 (22.6%), and active pulmonary inflammation in 22 (17.7%). There was no patient with diffuse pleural thickening or calcification in this study. Trivial, mild, and moderate emphysema was found in 31 (25.0%), 21 (16.9%), and 12 (9.7%) patients, respectively. Severe pleural adhesion was found in 31 (25.0%) patients. The association of localized pleural thickening or calcification on CT with severe pleural adhesion was not found (*P* = 0.405 and 0.107, respectively). Size of pulmonary calcified nodules and extent of emphysema were significant variables in a univariate analysis (*P* = 0.045 and 0.005, respectively). In a multivariate analysis, moderate emphysema was significantly associated with severe pleural adhesion (odds ratio of 11.202, *P* = 0.001). In conclusion, severe pleural adhesion might be found during lung cancer surgery, provided that preoperative chest CT shows substantial pulmonary calcified nodules or emphysema.

## Introduction

The presence of pleural adhesions at the start of video-assisted thoracoscopic surgery (VATS) or open thoracotomy inhibits the collapse of the lung and access to the pleural space and hilum [1, 2]. Consequent pleural injury, bleeding, and prolonged air leaks during adhesiolysis increase operation time and morbidity [3–5]. The prediction of pleural adhesion is necessary preoperatively to determine the appropriate surgical access and feasibility of the thoracoscopic approach. Several studies have tried to anticipate pleural adhesions with imaging modalities. Chest CT showed moderate value in predicting pleural adhesions, whereas a lot of patients with pleural adhesions demonstrated no pleural finding on CT [6, 7]. We assumed that other image characteristics on CT even in the lung parenchyma, which were suggestive of old inflammation or chronic injury, could be associated with severe pleural adhesion during surgery of lung cancer. Therefore, the purpose of this study was to investigate the association between image characteristics on preoperative chest CT and severe pleural adhesion in lung cancer patients who underwent VATS or open thoracotomy.

## Materials and Methods

### Study population

Included in this retrospective single-center analysis were consecutive patients who underwent thoracic surgeries for alleged or suspected lung cancer between January 2010 and December 2012. Any prior thoracic operation was an exclusion criterion. We investigated medical records to obtain baseline characteristics that included age, sex, height, weight, body mass index (BMI), current smoker, history of pulmonary tuberculosis or occupational disorders, such as silicosis, forced vital capacity (FVC), predicted percentage of forced expiratory volume in one second (FEV_1_% predicted), and FEV_1_/FVC. All patients underwent pulmonary function test (PFT) within 4 weeks prior to surgery.

### Ethics Statement

The study protocol was approved by Seoul National University_Boramae Hospital Institutional Review Board (16-2014-7). And the Institutional Review Board waived the need for written informed consent from the participants. All clinical investigation was conducted according to the principles expressed in the Declaration of Helsinki. Patient records were de-identified and analyzed anonymously.

### Preoperative chest CT protocol

CT was performed using a 16-channel multi-detector CT (MDCT) (Light-Speed, GE Healthcare) or a 64-channel MDCT (Brilliance; Philips Medical Systems, Cleveland, Ohio). CT scans were obtained with (and without) the administration of an intravenous contrast media. Unenhanced CT was performed from the thoracic inlet to the cardiac apex. Because pulmonary tuberculosis is endemic in our country, we have routinely performed addition unenhanced CT scans to differentiate calcification and enhancement in pulmonary nodules or lymph nodes in the thorax. CT with contrast enhancement was then performed from the carotid bulb level to the upper portion of the kidneys. For contrast-enhanced CT images, 80 to 120 mL of iopamidol (Iopamiro 300; Bracco, Milan, Italy) was administered intravenously at a rate of 2.5 mL/s. Helical CT data were acquired using a 16×1.5 mm or 64×0.625 mm collimation with a rotation speed of 0.5 or 0.42 s, a pitch of 1.11 to 1.25, and 120 kilovolt (peak). Effective milliampere-second ranged between 120 and 187 using an automatic tube current modulation technique. Transverse data sets were reconstructed with 2.5 mm thickness at 2.5 mm increments. Resultant images were transferred to a picture archiving and communication system (PACS) for image analysis. All CT scans were obtained within 4 weeks prior to surgery.

### Evaluation of preoperative chest CT findings

Two board-certified thoracic radiologists (blinded to the operative findings) retrospectively evaluated 2.5-mm thick axial preoperative chest CT images in consensus. They have 6 years`and 3 years`experience in thoracic radiology, respectively. The maximum diameter of the tumor was recorded. We also evaluated the presence of image findings suggestive of pleural invasion, such as an adjacent pleural thickening or soft tissue extending extrapleural fat layer.

The CT findings of pleural or pulmonary parenchymal inflammation or its sequel on the ipsilateral side of the surgery were included as image characteristics for the prediction of severe pleural adhesion: 1) pleural thickening or calcification, 2) pulmonary calcified nodules, 3) active pulmonary inflammation, 4) emphysema, 5) interstitial pneumonitis, and 6) bronchiectasis.

#### Pleural thickening or calcification

The pleural was defined as the layer of soft-tissue density at the chest wall–lung interface on the axial CT images. A pleural thick lining with >3 mm of maximum thickness with or without extrapleural fat proliferation was defined as pleural thickening [7]. Any calcification at the pleural area on the axial CT images was defined as pleural calcification. Calcification was defined as structures of attenuation above 130 Hounsfield unit (HU) on the unenhanced chest CT [8]. The extent of pleural thickening or calcification was visually estimated. We defined diffuse pleural thickening as a continuous sheet of pleural thickening more than 5 cm wide, more than 8 cm in craniocaudal extent, and more than 3 mm thick [9]. The extent of pleural thickening smaller than diffuse was defined as localized. Fibrothorax was defined as diffuse pleural thickening, narrowed intercostal spaces, a diminished size of the hemithorax, and retraction of the mediastinum toward the operated thorax with or without extrapleural fat proliferation [10].

#### Pulmonary calcified nodules

We recorded diameter and number of any calcified nodules in the lung parenchyma. A pulmonary calcified nodule was defined as a nodule containing any size of parenchymal calcification on the unenhanced CT images. We measured the maximum long diameter of each calcified nodule on axial CT image with mediastinal window setting (window width, 400 HU; window center, 30 HU). The size of pulmonary calcified nodules was defined as the added maximum long diameter (cm) of all pulmonary parenchymal calcified nodules in the operated thorax. We did not include lung cancer mass in the measurement if it contained dystrophic calcification.

#### Active pulmonary inflammation

We also recorded the presence of active inflammation in the lung parenchyma. CT finding of obstructive pneumonia or non-specific bronchiolitis such as consolidation or clustered ill-defined nodules in the ipsilateral operated thorax were defined as active pulmonary inflammation.

#### Emphysema, interstitial pneumonitis, and bronchiectasis

Emphysema was defined as the presence of areas of low attenuation that contrast surrounding lung parenchyma with normal attenuation [11]. The extent of emphysema was evaluated with a visual scoring system: 0 = no emphysema, 1 = < 5% (trivial), 2 = 5–25% (mild), 3 = 26–50% (moderate), 4 = 51–75% (severe), and 5 = > 76% involvement of both lungs (very severe) [12]. We used the same visual scoring system for emphysema to evaluate the extent of interstitial pneumonitis and bronchiectasis. To evaluate the overall extent of parenchymal abnormalities that suggested interstitial pneumonitis, we graded the extent of reticular opacity, honeycomb cysts, or ground glass opacity [13].

### Operative findings

Surgery was performed by one thoracic surgeon with 15 years of experience. We retrospectively investigated presence of any or severe pleural adhesion from the electrical medical records. We defined severe pleural adhesion, if pleural adhesion required sharp dissection and adhesiolysis for 30 minutes or longer. We divided study population into two groups; no or minimal pleural adhesion versus severe pleural adhesion.

### Statistical analysis

All data are expressed as mean ± standard deviation (SD) unless otherwise stated. We performed logistic regression analysis to examine the correlation of image characteristics with severe pleural adhesion. If image characteristics showed *P* value < 0.1 in simple logistic regression, we defined it as candidate variables related to severe pleural adhesion. To control the effects of possible confounding factors, simple logistic regression analyses were also performed for patient demographics such as age, gender, body mass index, as well as for history of pulmonary tuberculosis. If the possible confounding variables had a *P* value < 0.1, they were included as a covariant in the multiple logistic regression analyses. We conducted multiple logistic regression with backward elimination to avoid collinearity between variables. Furthermore, the Student t test and chi-square test was applied to evaluate differences in image characteristics between two groups. A *P* value of < 0.05 was considered statistically significant. Statistical analysis was performed with commercially available statistical software, SPSS version 20.0 (SPSS, Inc, an IBM Company, Chicago, Illinois, USA).

## Results

Among 125 consecutive patients who underwent thoracic surgeries for alleged or suspected lung cancer, one patient who has history of prior thoracic operation due to esophageal cancer was excluded. A total of 124 patients who underwent VATS (n = 91) or open thoracotomy (n = 33) were included in this study. Table 1 shows the baseline characteristics of patients. There was no significant difference between no severe pleural adhesion group and severe pleural adhesion group in baseline characteristics except the results of pulmonary function test. All data underlying the baseline characteristics and CT findings were uploaded as supporting information file (S1 Dataset).

**Table 1:** Patients’ characteristics.

Characteristics	Total (*n* = 124)	No severe pleural adhesion (n = 93)	Severe pleural adhesion (n = 31)	*P value*
Age	64.6 ± 10.4	63.8 ± 11.1	66.9 ± 7.4	0.071
Male gender*	93 (71.0)	68 (73.1)	25 (80.6)	0.479
Height	162.7 ± 8.1	162.7 ± 8.3	162.7 ± 7.9	0.977
Weight	62.3 ± 9.1	62.2 ± 9.3	62.7 ± 8.8	0.804
Body mass index	23.6 ± 3.2	23.7 ± 3.4	23.1 ± 2.3	0.362
Current smoker*	88 (71.0)	64 (68.8)	24 (77.4)	0.494
History of pulmonary tuberculosis*	22 (17.6)	13 (14.0)	9 (29.0)	0.100
History of occupational disorders such as silicosis or asbestosis*	0	0	0	n/a
FVC	3.3 ± 0.8	3.4 ± 0.8	2.9 ± 0.8	0.002
FEV_1_ predicted%	95.0 ± 20.1	99.8 ± 18.3	80.5 ± 18.9	< 0.001
FEV_1_/FVC%	69.7 ± 11.7	71.3 ± 10.3	64.6 ± 14.4	0.020

Abbreviations. FVC, Forced vital capacity; FEV_1_, Forced expiratory volume in 1 second, n/a not applicable.

Except where indicated, data are given as average value ± SD. SD, standard deviation. Numbers in parentheses are range.

*Data are given as numbers of subjects and numbers in parentheses are percentage.

Preoperative chest CT findings and presence of severe pleural adhesion in the operated thorax are described in Table 2. There was no patient with diffuse pleural thickening or calcification. Size of pulmonary calcified nodules was significant lower in no severe pleural adhesion group than in severe pleural adhesion group (0.3 ± 0.8 vs. 1.2 ± 3.2, *P* < 0.005). There was significant difference between two groups in terms of emphysema severity (*P* < 0.001). Moderate extent of emphysema was found in 4 patients (4.3%) in no severe pleural adhesion group and 8 (25.8%) in severe pleural adhesion group. Any pleural adhesion was found in 78 patients (62.9%) and severe pleural adhesion in 31 (25.0%). In patients who underwent VATS, any pleural adhesion was found in 52 patients (57.1%) and severe adhesion in 18 (19.8%). In patients who underwent open thoracotomy, any pleural adhesion was found in 26 patients (78.8%) and severe adhesion in 13 (39.4%).

**Table 2:** Preoperative chest CT findings in the operated thorax.

Characteristics	Total (*n* = 124)	No severe pleural adhesion (n = 93)	Severe pleural adhesion (n = 31)	*P value*
Size of tumor (cm)*	3.3 ± 1.7	3.3 ± 1.6	3.3 ± 1.8	0.995
Pleural invasion of tumor	40 (32.3)	30 (32.3)	10 (32.3)	1.000
Localized pleural thickening	8 (6.5)	5 (5.4)	3 (9.7)	0.411
Localized pleural calcification	8 (6.5)	4 (4.3)	4 (12.9)	0.107
Diffuse pleural thickening or calcification	0	0	0	n/a
Fibrothorax	0	0	0	n/a
Pulmonary calcified nodules	28 (22.6)	18 (19.4)	10 (32.3)	0.145
Size of pulmonary calcified nodules (cm)*	0.5 ± 1.7	0.3 ± 0.8	1.2 ± 3.2	0.005
Active pulmonary inflammation	22 (17.7)	13 (14.0)	9 (29.0)	0.100
Emphysema				0.001
none	60 (48.4)	52 (55.9)	8 (25.8)	
< 5% (trivial)	31 (25.0)	22 (23.7)	9 (29.0)	
5–25% (mild)	21 (16.9)	15 (16.1)	6 (19.4)	
26–50% (moderate)	12 (9.7)	4 (4.3)	8 (25.8)	
> 50% (severe)	0	0	0	
Interstitial pneumonitis				0.077
none	115 (92.7)	89 (95.7)	26 (83.9)	
< 5% (trivial)	6 (4.8)	3 (3.2)	3 (9.7)	
5–25% (mild)	0	0	0	
26–50% (moderate)	3 (2.4)	1 (1.1)	2 (6.5)	
> 50% (severe)	0	0	0	
Bronchiectasis				0.289
none	113 (91.1)	83 (89.2)	30 (96.8)	
< 5% (trivial)	0	0	0	
5–25% (mild)	11 (8.9)	10 (10.8)	1 (3.2)	
> 50% (severe)	0	0	0	

Abbreviations. n/a not applicable.

Except where indicated, data are given as numbers of subjects and numbers in parentheses are percentage.

*Data are given as average value ± SD. SD, standard deviation. Numbers in parentheses are range.

**Fig 1:**
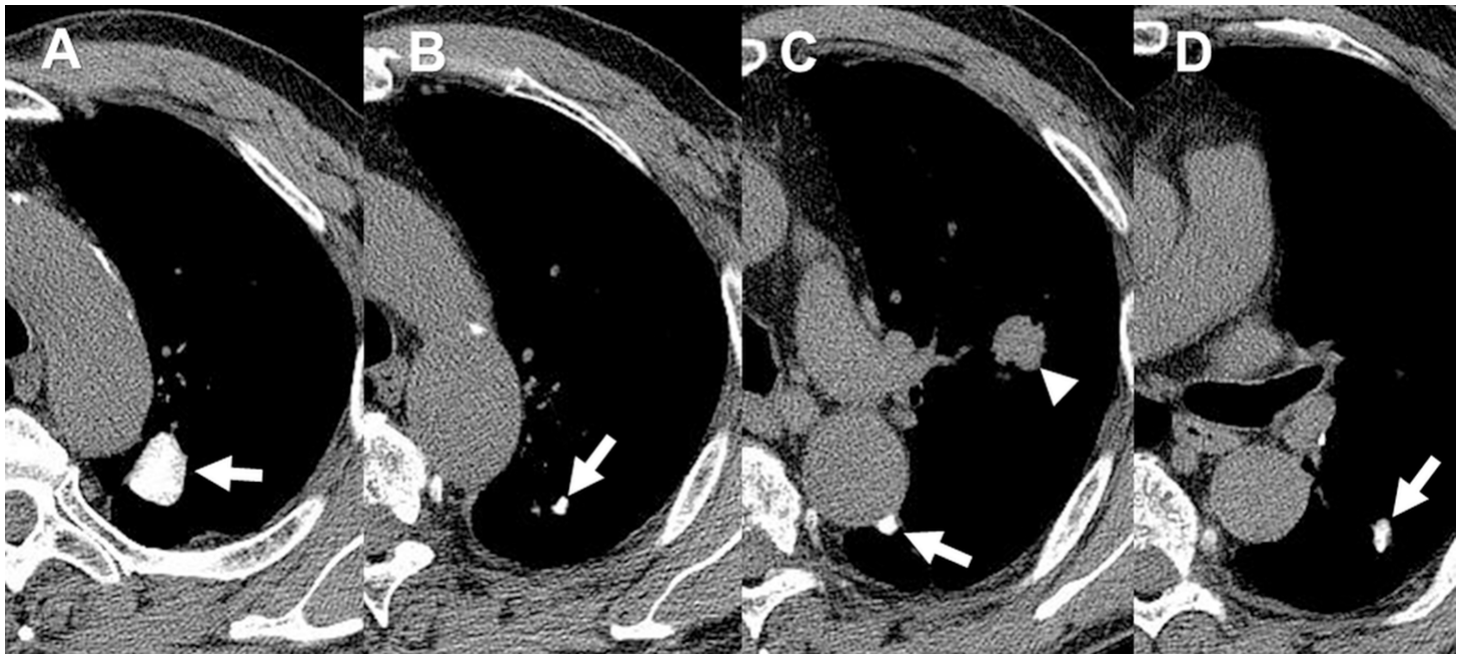
A 78-year-old men with squamous cell carcinoma in the left upper lobe. (A-D) Non-contrast-enhanced chest CT images demonstrate multiple calcified nodules in the left lung (arrows). (C) CT image demonstrates a nodule (arrowhead) in the left upper lobe. He underwent left upper lobectomy and systemic nodal dissection via thoracotomy. During the operation, there was severe pleural adhesion requiring seven hours for adhesiolysis.

In univariate analysis, size of pulmonary calcified nodules and pulmonary emphysema were significant variables related to severe pleural adhesion (Table 3) (Fig 1).

**Table 3:** The results of logistic regression analyses to evaluate the association between the image characteristics on chest CT and severe pleural adhesion in lung cancer patients.

Variables	Severe pleural adhesion					
	Simple			Multiple		
	OR	95% CI	*P* value	OR	95% CI	*P* value
Age	1.034	0.989–1.081	0.137			
Male gender	0.653	0.240–1.778	0.404			
BMI	0.939	0.822–1.074	0.359			
Current smoking	1.618	0.628–4.168	0.319			
History of pulmonary tuberculosis	2.517	0.952–6.655	0.063			
Pleural invasion of tumor	1.000	0.419–2.386	1.000			
Localized pleural thickening	1.886	0.424–8.394	0.405			
Localized pleural calcification	3.296	0.772–14.070	0.107			
Pulmonary calcified nodules	1.987	0.797–4.939	0.141			
Size of pulmonary calcified nodules	1.397	1.007–1.939	0.045	1.377	0.956–1.983	0.086
Active pulmonary inflammation	2.517	0.952–6.655	0.063	2.525	0.839–7.599	0.099
Emphysema			0.005			0.014
< 5% (trivial)	2.659	0.908–791	0.075	2.212	0.734–6.670	0.159
5–25% (mild)	2.600	0.780–8.670	0.120	1.951	0.501–7.606	0.335
26–50% (moderate)	13.000	3.167–53.370	< 0.001	11.202	2.600–48.267	0.001
Bronchiectasis	0.277	0.034–2.254	0.230			
Interstitial pneumonitis			0.117			
< 5% (trivial)	3.423	0.652–17.983	0.146			
26–50% (moderate)	6.846	0.597–78.538	0.122			

Abbreviations. OR, odds ratio; CI, Confidence interval; BMI, Body mass index; FEV_1_, Forced expiratory volume; FVC, Forced vital capacity.

**Fig 2:**
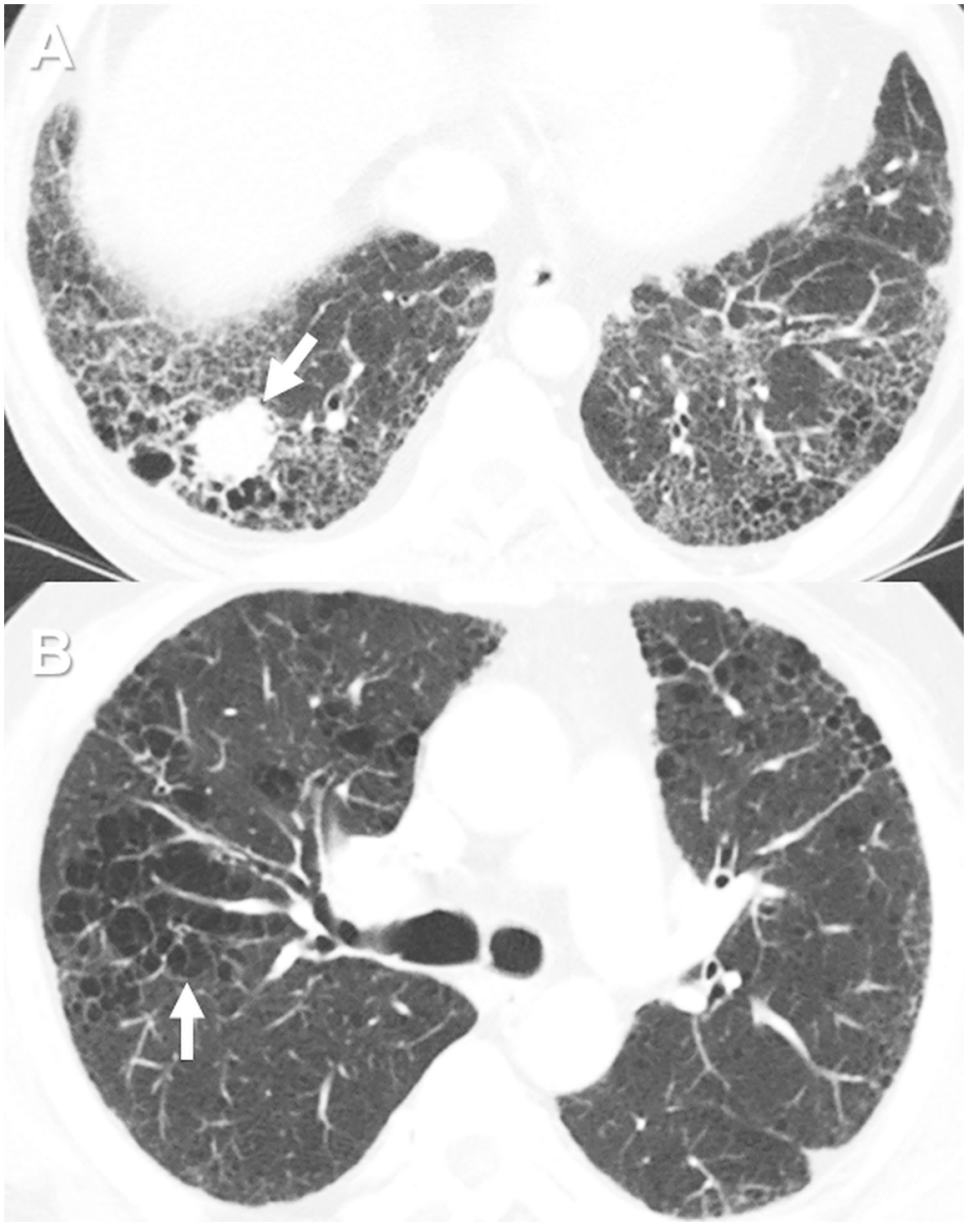
A 61-year-old men with squamous cell carcinoma in the right lower lobe. (A) Axial CT image with lung window setting demonstrates a 2.5-cm nodule in the right lower lobe (arrow). Reticular opacities in the base of both lower lobes were noted, suggesting interstitial pneumonitis (B) CT image at the level of carina demonstrates multiple round low attenuating areas in both lungs (arrow), suggesting emphysema. He underwent right lower lobectomy and systemic nodal dissection via video assisted thoracoscopic surgery. During the operation, there was severe pleural adhesion requiring nine hours for adhesiolysis.

Multiple logistic regression analysis showed that moderate emphysema was significantly associated with severe pleural adhesion (odds ratio of 11.202, *P* = 0.001) (Fig 2). Moderate emphysema was found in 8 patients (25.8%) in severe pleural adhesion group and 4 (4.3%) in no or minimal adhesion group. As the extent of emphysema was higher, severe pleural adhesion was more frequent (*P* < 0.001); 13.3% (n = 8) for none, 29.0% (n = 9) for < 5%, 28.6% (n = 6) for 5–25%, and 66.7% (n = 8) for 26–50%, respectively.

## Discussion

In previous studies, including patients with benign or malignant thoracic disease, pleural adhesion during VATS or open thoracotomy was reported in a wide range; 38.5%, 60.9%, and 83.0% for any pleural adhesion [7, 14, 15] and 5.5% for severe pleural adhesion [16]. For lung cancer patients who underwent VATS lobectomy, it was reported that any pleural adhesion and moderate or severe adhesion were found in 51.7% and 11.7%, respectively [17], which was similar to the results of our study (57.1% for any pleural adhesion and 19.8% for severe pleural adhesion in VATS group).

With the use of transthoracic ultrasonography, the presence and location of a pleural adhesion prior to a thoracic operation can be identified with a high negative predictive value [14, 18]. However, it is time-consuming and requires technically experienced examiners. In a previous study, chest CT showed low sensitivity (38.0%) and specificity (46.0%) to the identification of pleural adhesion on a lesion-by-lesion analysis of patients who underwent VATS [7]. We analyzed our CT data on a patient-by-patient basis and recorded the presence of severe pleural adhesion in each patient. In multivariate analysis, we revealed that moderate emphysema on chest CT significantly associated with severe pleural adhesion (odds ratio of 11.202, *P* = 0.001). The presence of pleural adhesion in emphysema patients who underwent lung volume reduction surgeries has been reported with a variable range of incidence (41.3%, 50.8%, and 80.0%) [3, 19, 20]. In our study, emphysema was found in 51.6%. As the extent of emphysema was higher, severe pleural adhesion was more frequent. In patient with moderate emphysema, severe pleural adhesion was found in 66.7%. To our knowledge, this study is the first to report showing the association of emphysema revealed on CT with severe pleural adhesion. Pulmonary emphysema is main CT finding of chronic obstructive pulmonary disease [21]. Patients with chronic obstructive pulmonary disease are at a higher risk of developing community-acquired pneumonia than patients in the general population [22–24]. And chronic obstructive pulmonary disease severity is associated with severe pneumonia [25]. Therefore we can assume that possible pneumonia and associated pleural inflammation in patients with severe emphysema can make subsequent severe pleural adhesion.

Diffuse pleural thickening or calcification caused by asbestos, hemothorax, or bacterial or tuberculous empyema is known to be associated with severe pleural adhesion [26]; however, in our study, localized pleural thickening or calcification was not associated with severe pleural adhesion (*P* = 0.405 for localized pleural thickening and *P* = 0.107 for localized pleural calcification). It is different from the results of previous study that showed a moderate value of pleural thickening on CT [7]. The reason for the discordance may reflect differences in the patient populations which were studied. Mason et al. reported that 44.4% (n = 28) of patients showed pleural change on CT suggesting pleural adhesion among 63 patients [7]. In contrast, there were only 2 patients with both pleural thickening and calcification in the operated thorax in this study with no patient with diffuse pleural thickening or calcification on CT.

In our study, 24 patients (77.4%) among 31 with severe pleural adhesion showed no pleural change on CT. It is in agreement with previous studies showing pleural adhesion without any pleural finding on CT [7, 27]. And there might be false detection of pleural thickening on CT. The thick soft-tissue density at the chest wall–lung interface on the axial CT images sometimes do not truly suggest pleural thickening on CT. Physiological pleural fluid accumulation or dependent atelectasis can mimic the presence of pleural thickening or enhancement on CT [28].

Pulmonary calcified nodules are often the result of dystrophic calcification in areas of an injured lung following an inflammatory process, such as infection, bleeding, or pulmonary infarction [29, 30]. Infectious diseases are the most common cause of calcified nodules [31, 32]. A univariate analysis revealed the size of pulmonary calcified nodules was significantly associated with severe pleural adhesion (odds ratio of 1.397, *P* = 0.045); consequently, the possibility of severe pleural adhesion increased as the size of pulmonary calcified nodules increase. It is likely the size of pulmonary calcified nodules is proportional to the severity of lung injury and associated pleural inflammation.

This study has several limitations. First, we retrospectively selected patients with the same ethnic background and living in the same geographic region; therefore, the results of this study should be interpreted cautiously. Second, the presence and severity of pleural adhesion was evaluated mainly in terms of time needed for adhesiolysis. We did not compare and match the location of intraoperative pleural adhesion and CT findings. Third, CT analysis was performed with consensus readings. We did not measure the variability between the reviewers. Fourth, CT findings, such as size of pulmonary calcified nodules or severity of emphysema or interstitial fibrosis, were visually estimated and semi-quantified. Semi quantification of CT findings can affect the reproducibility of our results.

In conclusion, severe pleural adhesion might be found during lung cancer surgery, provided that preoperative chest CT shows substantial pulmonary calcified nodules or emphysema.

## Supporting Information

S1 Dataset De-identified dataset. Clinical and computed tomographic data of all patients were included with file format of Microsoft Excel.
(XLSX)
